# Influence of a prudent diet on circulating cathepsin S in humans

**DOI:** 10.1186/1475-2891-13-84

**Published:** 2014-08-16

**Authors:** Elisabeth Jobs, Viola Adamsson, Anders Larsson, Magnus Jobs, Elisabet Nerpin, Erik Ingelsson, Johan Ärnlöv, Ulf Risérus

**Affiliations:** Department of Public Health and Caring Sciences/Geriatrics, Uppsala University, Uppsala, Sweden; Department of Public Health and Caring Sciences, Clinical Nutrition and Metabolism, Uppsala University, 75185 Uppsala Science Park, Kragujevac, Sweden; Dalarna University, School of Health and Social Studies, Falun, Sweden; Department of Medical Sciences, Uppsala University, Uppsala, Sweden

**Keywords:** Nordic prudent diet, Cathepsin S, Weight loss, Cardiometabolic risk factors

## Abstract

**Background:**

Increased circulating cathepsin S levels have been linked to increased risk of cardiometabolic diseases and cancer. However, whether cathepsin S is a modifiable risk factor is unclear. We aimed to investigate the effects of a prudent diet on plasma cathepsin S levels in healthy individuals.

**Findings:**

Explorative analyses of a randomized study were performed in 88 normal to slightly overweight and hyperlipidemic men and women (aged 25 to 65) that were randomly assigned to *ad libitum* prudent diet, i.e. healthy Nordic diet (ND) or a control group (habitual Western diet) for 6 weeks. Whereas all foods in the ND were provided, the control group was advised to consume their habitual diet throughout the study. The ND was in line with dietary recommendations, e.g. low in saturated fats, sugars and salt, but high in plant-based foods rich in fibre and unsaturated fats.

The ND significantly decreased cathepsin S levels (from 20.1 (+/-4.0 SD) to 19.7 μg/L (+/-4.3 SD)) compared with control group (from 18.2 (+/-2.9 SD) to 19.1 μg/L (+/-3.8 SD)). This difference remained after adjusting for sex and change in insulin sensitivity (P = 0.03), and near significant after adjusting for baseline cathepsin S levels (P = 0.06), but not for change in weight or LDL-C. Changes in cathepsin S levels were directly correlated with change in LDL-C.

**Conclusions:**

Compared with a habitual control diet, a provided *ad libitum* healthy Nordic diet decreased cathepsin S levels in healthy individuals, possibly mediated by weight loss or lowered LDL-C. These differences between groups in cathepsin S were however not robust and therefore need further investigation.

## Findings

### Introduction

Cathepsin S is highly expressed in antigen presenting cells [1–3] and has important functions in the major histocompatibility complex (MHC) class II antigen presentation [4]. Furthermore it degrades extracellular matrix [5].

Elevated circulating cathepsin S concentrations predict mortality in elderly men [6] and it has been linked to cardiovascular disease (CVD), type 1 and type 2 diabetes, cancer [7–10] and inflammation [11, 12]. Cathepsin S is also associated with LDL-C and HDL-C [13–15]. Furthermore it is increased in adipose tissue and serum of obese persons [16, 17], whereas studies in obese women have indicated that weight loss by obesity surgery or energy-restricted diet decrease serum cathepsin S [17, 18].

As cathepsin S appears to be a novel promising risk marker of both cardiometabolic and malignant diseases, it is important to examine the effect of diet on cathepsin S concentrations. We hypothesized that a healthy nordic diet (ND) could reduce plasma levels of cathepsin S. The aim was to investigate the effects of a ND, eaten *ad libitum*, on plasma levels of cathepsin S in the NORDIET-trial. A secondary aim was to investigate the relationships between changes in cathepsin S concentrations and changes in cardiometabolic risk factors.

### Subjects and methods

#### Subjects

During December 2007, subjects living in Bollnäs, Sweden, were recruited by advertisements in the local newspaper. The inclusion criteria were healthy (as assessed by a physician), men and women between 25 and 65 years, plasma LDL-C ≥3.5 mmol/L¯^1^, body mass index (BMI) ≥20 and ≤ 31 kg m^2^, for women and men respectively [19]. Subjects with hypertension, CVD, diabetes and other chronic disease and those on lipid-lowering drugs were excluded.

#### Study design

The study was conducted between February and May, 2008. Eighty-eight subjects were randomly assigned to one of two groups: a ND or a control group following their usual diet.

Clinical and laboratory assessments were performed at baseline and after 6 weeks. The trial was conducted in accordance with the CONSORT statement and registered in the Current Controlled Trials database (http://www.controlled-trials.com); International Standard Randomized Controlled Trial Number (ISRTCTN): 77759305.

Written informed consent was given by all subjects. The study was approved by the regional ethical committee in Uppsala.

#### Outcome measures

In the present post-hoc study of the NORDIET-trial we aimed to investigate changes in plasma cathepsin S levels during ND as compared with a control group. We also investigated possible relationships between change in plasma cathepsin S and changes in weight, insulin sensitivity, triglycerides (TG), LDL-C, HDL-C, systolic blood pressure (SBP) and diastolic blood pressure (DBP).

#### Intervention

All main meals were provided to the subjects in the ND group. The assessment of diet and the change in diet during the study has been described previously [19].

#### Prudent diet

The ND was based on the Nordic nutrition recommendations (NNR) [20] and contained characteristic foods used in Nordic countries including fruits (e.g. apples) and berries (e.g. blueberries), legumes, vegetables, low-fat dairy products and fatty fish (e.g. salmon). The ND also included LDL-C lowering foods (e.g. oats; barley, almonds and psyllium seeds) [21, 22]. The ND was provided *ad libitum*.

#### Control group

The subjects in the control group were instructed to follow their habitual diet, eaten ad libitum, and continue their usual physical activity.

#### Clinical assessment

Bodyweight was measured (kg) in light clothing without shoes, on a digital scale. Blood pressure was measured manually in a sitting position after 5 minutes rest. Two measurements were performed with a 2 minutes interval, and the average value was calculated.

#### Biochemical analysis

Venous blood was collected after a 12 h fast and plasma separated and frozen in -70°C before analyses. Plasma cathepin S was measured by ELISA (human cathepsin S (Total), DY1183, R&D Systems). The intraassay CV was 7%. Glucose, total cholesterol, TG and HDL-C plasma concentrations were measured using a Roche Diagnostics Cobas® 6000. Plasma LDL-C was calculated by Friedewalds formula [23]. Plasma insulin was measured by an enzyme-linked immunoassay kit (Mercodia AB, Uppsala, Sweden). Homeostasis model assessment-insulin resistance (HOMA-IR) was calculated [24].

#### Statistical analysis

Data are presented as mean ± SD. Per protocol analysis was used to assess effects of diet on outcome measures. Unpaired T-test was used to assess differences in plasma levels of cathepsin S during follow up, between the two groups, and as a second step, we used ANCOVA to adjust for sex, weight and cardiometabolic risk factors. To assess associations between change in cathepsin S and change in cardiometabolic risk factors linear regression and Pearson’s correlation was used. A two-tailed P-value of 0.05 was regarded as significant. STATA, version 11 was used for statistical analysis.

## Results

Only two subjects (one in each group) dropped out [19], leaving 86 subjects with data on cathepsin S. After randomization, the two groups were almost identical with regards to the baseline characteristics, with the exception of a significant difference in cathepsins S levels between the groups (Table 1).

**Table 1:** **Baseline characteristics after randomization**

Characteristics	Control group	Healthy Nordic diet	P-value
**Subjects, n**	42	44	
**Age (year)**	53.4 ± 8.1	52.6 ± 7.8	0.63
**Men/women**	15/27	17/27	0.83
**Body weight (kg)**	78.0 ± 13.3	76.0 ± 10.5	0.44
**Body mass index (kg m¯** ^**2**^ **)**	26.5 ± 3.3	26.3 ± 3.2	0.79
**SBP (mmHg)**	123 ± 14	128 ± 12	0.50
**DBP (mmHg)**	83 ± 9	81 ± 7	0.16
**Plasma TG (mmol/L¯** ^**1**^ **)**	1.4 ± 0.8	1.6 ± 0.8	0.32
**Plasma cholesterol (mmol/L¯** ^**1**^ **)**	6.4 ± 0.7	6.2 ± 0.8	0.36
**Plasma LDL cholesterol (mmol/L¯** ^**1**^ **)**	4.2 ± 1.0	4.0 ± 0.6	0.33
**Plasma HDL cholesterol (mmol/L¯** ^**1**^ **)**	1.5 ± 0.5	1.5 ± 0.4	0.28
**LDL/HDL ratio**	2.8 ± 1.0	2.9 ± 0.8	0.80
**Plasma glucose (mmol/L¯** ^**1**^ **)**	4.9 ± 0.6	4.9 ± 0.5	0.54
**Insulin resistance (HOMA-IR)**	1.3 ± 0.6	1.2 ± 0.6	0.47
**Cathepsin S μg/L**	18.3 ± 2.99	20.1 ± 4.1	0.02

Data are means ± SD. HDL: High-density lipoprotein; LDL: Low-density lipoprotein; HOMA-IR: Homeostasis model assessment-insulin resistance; SBP: systolic blood pressure; DBP: diastolic blood pressure; TG: triglycerides. Differences between the Nordic Diet and Control groups were assessed using unpaired two-tailed *t*-tests.

### Effect of diet on cathepsin S

**Figure 1:**
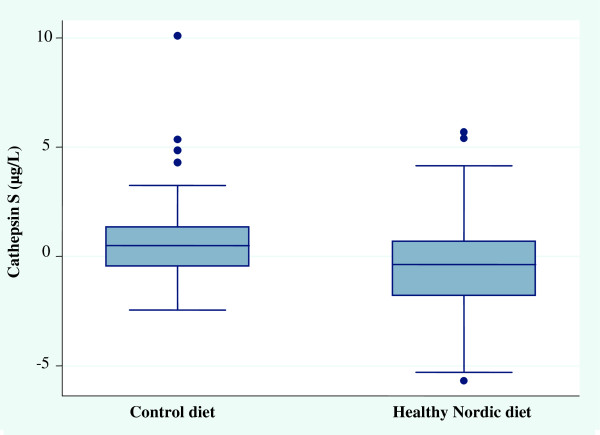
**Differences in serum cathepsin S levels between the control diet and healthy Nordic diet (ND) from baseline to 6 weeks.** Serum levels of cathepsin S were decreased compared with the control diet (P = 0.03).

As previously reported [19], ND reduced body weight (mean 3 kg) as compared with the control group. Here we report a significant difference between ND and control group in the change of cathepsin S concentrations (P = 0.03 for between group difference, Figure 1).

The ND decreased plasma cathepsin S levels, from 20.1 (+/-4.05 SD) to 19.7 μg/L (+/-4.3 SD) whereas the control group changed from 18.2 (+/-2.9 SD) to 19.1 μg/L (+/-3.8 SD) (Figure 1). Mean difference in change between groups were 12.0 μg/L (+/-25.2 SD). These differences remained after adjusting for sex (p = 0.03) and insulin sensitivity (P = 0.03), whereas adjusting for weight change (P = 0.22), LDL-C (P = 0.36) and total cholesterol (P = 0.48) abolished the significant difference between groups. However, adjusting for baseline cathepsin S levels attenuated the difference between groups (p = 0.06), as well as adjustments for differences in systolic and diastolic blood pressure (p = 0.06). As described previously [19], dietary compliance was overall excellent and the dietary goals were achieved for most nutrients”. E.g. total fat was 27%E (goal 25-35%E), carbohydrate 52%E (45-60%E), protein 19%E (10-20%E), fibre 54%E (25–35) and saturated fat 5%E (<8%E).

### Correlations between changes in cathepsin S and cardiometabolic risk factors

Changes in cathepsin S tended to be correlated with changes in weight (P = 0.05), which remained after adjusting for sex (P = 0.04). Furthermore, change in cathepsin S were correlated with change in LDL-C (P = 0.03) and total cholesterol (P = 0.01). These associations remained significant after adjusting for sex (P = 0.03 and 0.01 respectively). Change in cathepsin S did not correlate with changes in insulin sensitivity, TG, HDL-C, SBP or DBP (Table 2).

**Table 2:** **Correlations between change in serum cathepsin S concentrations and changes in weight and cardiometabolic risk factors during 6 weeks in the whole sample (n = 86)**

Characteristics	r	P-value	β-coefficient (95% CI)	P-value
Weight	0.22	0.04	241.60 (3.37-479.81)	0.05
HOMA-IR	0.04	0.72	166.02 (-734.40-1066.44)	0.72
LDL-C	0.24	0.03	794.11 (84.86-1503.36)	0.03
HDL-C	0.18	0.12	1914.54 (-505.34-4334.43)	0.12
TG	0.02	0.84	114.98 (-1007.58-1237.56)	0.84
Cholesterol	0.27	0.01	755.29 (163.79-1346.90)	0.01
SBP	0.15	0.18	29.43 (-13.37-72.24)	0.18
DBP	0.17	0.12	46.16 (-12.39-104.71)	0.12

Data are correlation coefficients and regression coefficients, with 95% CI. All models are adjusted for sex. HOMA -IR: Homeostasis model assessment-insulin resistance, HDL-C:High-density lipoprotein cholesterol; LDL -C: Low-density lipoprotein cholesterol, TG: Triglycerides, SB P: Systolic blood pressure, DBP: Diastolic blood pressure; Pearson's correlation and Linear regression has been used.

## Discussion

Adherence to an *ad libitum* ND for 6 weeks slightly decreased levels of plasma cathepsin S in normal or slightly overweight individuals, compared with the control group. Change in circulating cathepsin S concentrations were correlated with changes in body weight, LDL-C and total cholesterol suggesting that these factors may mediate the effect on cathepsin S levels.

To our knowledge, there are no studies investigating the effects of a prudent diet on cathepsin S concentrations. In accordance with our results, studies in obese women, showed that energy restriction and weight loss reduced cathepsin S mRNA and cathepsin S release in adipose tissue as well as serum levels [17, 18]. Body weight decreased by on average 3 kg during ND [19], and there was a near significant correlation between the change in body weight and change in cathepsin S. This study suggests that an *ad libitum* diet reduces weight and cathepsin S levels also in non-obese subjects, including men. The decreased cathepsin S levels did not remain significant after adjusting for weight change, suggesting that weight reduction mediated some of the dietary effect on cathepsin S. Adjusting for baseline levels of cathepsin S resulted in a P-value of 0.06 which may indicate lack of statistical power, rather than a lack of effect on cathepsin S. However, regression-towards-the mean effect cannot be completely excluded. Cathepsin S is strongly associated with cardiovascular risk factors, such as elevated triglycerides [17] and LDL-C [13]. Subjects on ND for 6 weeks markedly improved their cardiovascular risk profile, including lowering of LDL-C, insulin resistance, and blood pressure [19] and it is possible that this improvement affected the levels of cathepsin S. Our study supports such findings since adjustment for change in LDL-C and total cholesterol as well as change in body weight abolished the significant difference between the groups.

Higher levels of circulating cathepsin S are associated with insulin sensitivity [10]. In the current study we could not find a correlation between changes in serum cathepsin S and changes in HOMA-IR. However, the change in insulin sensitivity induced by the 6-week ND was moderate, albeit statistically significant [19]. Perhaps the discrepancy also could be explained by insulin sensitivity being measured with euglycemic clamp in the observational study, whereas it was estimated by HOMA-IR in the present study.

The strengths of this study include the randomized controlled design, and also all foods were provided to the ND group ensuring high compliance and low drop-out rates. It should however be noted that some of the observed effect on cathepsin S levels was likely to be caused by the fact that all foods was provided to the ND, but not the control group. Also, these results cannot be directly translated to clinical settings were dietary advice alone is given. Further studies are needed where all foods are provided to both ND and control groups, or dietary advice is given to both groups. Although all subjects in both groups were instructed to maintain their physical activity level during the study, possible differences between groups in physical activity level and smoking were not assessed and may thus have introduced some bias. Since only Caucasian Swedish subjects were included, generalizability to other ethnic groups is unclear. Further, it should be noted that this is a post-hoc study of the NORDIET trial, and thus there was no power calculation done on cathepsin S when designing this trial.

## Conclusion

These results suggest that a prudent diet comprising healthy Nordic foods may moderately reduce cathepsin S levels in non-obese men and women. This association seems to be partly, mediated by diet-induced weight loss and/or reduced LDL-C concentrations. Given the close link between cathepsin S and various obesity-linked diseases and increased mortality risk, the present results warrant further investigation in further studies.

## Abbreviations

ANCOVA: Analysis of covariance
BMI: Body mass index
CVD: Cardiovascular disease
DBP: Diastolic blood pressure
HOMA-IR: Homeostasis model assessment-insulin resistance
LDL-C: Low density lipoprotein cholesterol
MHC: Major histocompatibility complex
ND: Nordic diet
NNR: Nordic Nutrition recommendations
SBP: Systolic blood pressure
TG: Triglycerides.
